# Lipids or Proteins: Who Is Leading the Dance at Membrane Contact Sites?

**DOI:** 10.3389/fpls.2019.00198

**Published:** 2019-02-21

**Authors:** Jules D. Petit, Françoise Immel, Laurence Lins, Emmanuelle M. Bayer

**Affiliations:** ^1^UMR5200 CNRS, Laboratory of Membrane Biogenesis, University of Bordeaux, Villenave d’Ornon, France; ^2^Laboratoire de Biophysique Moléculaire aux Interfaces, TERRA Research Centre, GX ABT, Université de Liège, Liège, Belgium

**Keywords:** membrane contact sites, plants, lipids, tether proteins, plasmodesmata, biophysics

## Abstract

Understanding the mode of action of membrane contact sites (MCSs) across eukaryotic organisms at the near-atomic level to infer function at the cellular and tissue levels is a challenge scientists are currently facing. These peculiar systems dedicated to inter-organellar communication are perfect examples of cellular processes where the interplay between lipids and proteins is critical. In this mini review, we underline the link between membrane lipid environment, the recruitment of proteins at specialized membrane domains and the function of MCSs. More precisely, we want to give insights on the crucial role of lipids in defining the specificity of plant endoplasmic reticulum (ER)-plasma membrane (PM) MCSs and we further propose approaches to study them at multiple scales. Our goal is not so much to go into detailed description of MCSs, as there are numerous focused reviews on the subject, but rather try to pinpoint the critical elements defining those structures and give an original point of view by considering the subject from a near-atomic angle with a focus on lipids. We review current knowledge as to how lipids can define MCS territories, play a role in the recruitment and function of the MCS-associated proteins and in turn, how the lipid environment can be modified by proteins.

## Introduction

From an evolutionary perspective, membrane contact sites (MCSs) have been suggested to be the first contacts between archeon and protobacterium, leading to the emergence of eukaryotic cells (Jain and Holthuis, 2017). More generally, MCSs are described as a very close apposition (10–30 nm gap) of membranes of usually two different organelles (intra-organellar MCSs also exist), with specific lipid and protein populations (Bayer et al., 2017; Wang et al., 2017). MCSs create micro-environments that are under tight spatial and temporal control. Their main function is to promote fast inter-organellar communication through direct exchange of molecules such as lipids or calcium and through coordinated actions, for instance, with proteins acting in *trans* on the adjacent membrane to control receptor signaling or lipid synthesis (Eden et al., 2010; Haj et al., 2012; Himschoot et al., 2017; Muallem et al., 2017; Henrich et al., 2018). MCSs’ capacity to create and modulate micro-environments but also macro-environment at larger scales in the cell, is determined by high regulation of lipids and proteins, both in composition and distribution (Eisenberg-Bord et al., 2016; Gatta and Levine, 2017; Muallem et al., 2017). Many research have been made on the diversity of membrane lipids and the consequences of their heterogeneous distributions along and across the bilayer (Cacas et al., 2016; Sezgin et al., 2017; Gronnier et al., 2018; Harayama and Riezman, 2018). There is also increasing knowledge about the identity and function of MCS-associated proteins (Eisenberg-Bord et al., 2016; Wong et al., 2018). The exact definition of the MCSs is still being discussed but an emerging consensus is that they are (1) involved in the bulk lipid distribution and/or the fine regulation of membrane lipid composition through (but not only) direct lipid transfer which in turn is critical for local and organellar cellular processes and (2) characterized with the presence of tethering elements to hold the membranes close to each other but without undergoing fusion. Lipid transfer proteins (LTPs) are locally found at MCSs and, in addition to lipid transfer, some are also able to act as tethers (Lahiri et al., 2015; Eisenberg-Bord et al., 2016; Quon et al., 2018; Tong et al., 2018). In turn, the lipids are one of the main actors for LTP/tether recruitment, hence stability and function of MCSs (Bian et al., 2018; Wong et al., 2018). In such an environment, it is challenging to understand the dynamics and relationships between proteins and lipids but also interactions between lipid-lipid and protein-protein inside these confined areas filled with such a dynamic complexity.

We chose here to give a global view and additional thoughts on the role of lipids at plant MCSs, mainly at endoplasmic reticulum (ER)-plasma membrane (PM) MCSs (EPCSs). In this review, we will first describe the different ways lipids can define specific regions and regulate protein complexes through the formation of lipid domains, the regulation of membrane curvature and membrane electrostatics. Secondly, we will look at the importance of lipid exchange at MCSs. Thirdly, we will open a discussion about the particularity of plasmodesmata MCSs and their potential implications in organelle crosstalk, cell-to-cell communication and trafficking regulation. Finally, we list a number of multidisciplinary approaches that could be used to provide a complete view of these structures at (near) atomic and molecular levels.

## Membrane Lipids Create Unique Environments That Define and Regulate Mcss

MCSs have specific molecular compositions in both lipids and proteins, which define nano- and microdomains within the organelle. These subdomains are very important for the cellular polarization of signaling events via the formation of protein complexes, notably receptor complexes that are as such spatially and temporally regulated, driving acute signaling pathways (Burkart and Stahl, 2017; Gronnier et al., 2018). The molecular mechanisms leading to subcompartmentalization in general terms are gradually being uncovered and have been shown to involve lipids, membrane biophysical properties and the concerted action of specific protein machineries. Membrane subdivision is arising from the combination of membrane biophysical properties – such as fluidity, thickness, curvature and electrostatics – and has consequences in the recognition pattern of a plethora of lipid environment-sensing protein domains (Prévost et al., 2015; Strahl et al., 2015; Pérez-Lara et al., 2016; Lorent et al., 2017; Platre et al., 2018; Wong et al., 2018).

### Membrane Fluidity and Domains

**FIGURE 1:**
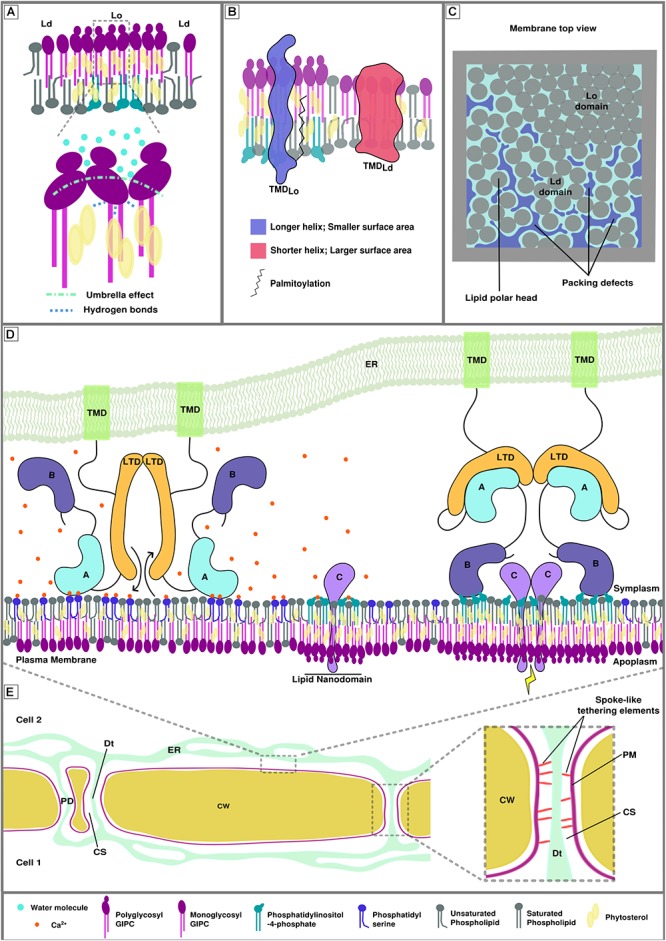
Membrane biophysical properties and lipid-protein interplay at membrane contact sites (MCSs). **(A)** Poly glycosylated GIPCs tend to increase the size and rigidity of phytosterol-dependent ordered membrane domains (Lo) through hydrogen bonding between the hydroxyl group of the sterols and the polarized groups of the GIPCs located at the polar/hydrophobic interface. This interaction is also favored by the umbrella effect of the big GIPCs’ polar moiety, which prevents water molecules to interact deeper into the bilayer (Grosjean et al., 2015). **(B)** Transmembrane protein distribution between different lipid domains relies on transmembrane length, surface area and palmitoylation (adapted from Lorent et al., 2017). **(C)** Representation of the lipid packing of membrane domains. Liquid ordered domain are more tightly packed than liquid disordered domains (Ld) because of the nature of the lipids and degree of their acyl chain saturation. Lipid packing defects arise in liquid disordered domains. **(D)** Hypothetical model of calcium-dependent regulation of protein-plasma membrane interaction at endoplasmic reticulum-plasma membrane MCS (EPCS). This hypothetical model gathers the possible interactions involving proteins, lipids and ions that could occur at MCS during signaling events. Its goal is to illustrate the complexity of lipid/protein/ion interactions. The protein illustrated here represents a lipid transfer protein/tether element that specifically locates to EPCS upon homodimerization. ***Left.*** In presence of calcium, domain A is able to interact with phosphatidylserine, the inter-membrane gap is reduced, allowing the exchange of lipids by the lipid transfer domains (LTDs). Domain B cannot interact with the phosphatidylinositol phosphates of the lipid nanodomains as they are shielded by the calcium ions **(*Middle*)**. ***Right.*** In the absence of calcium, domain A is released from the membrane, increasing the inter-membrane gap, and binds to the LTD, inhibiting lipid exchange between organelles. Domain B docks onto the lipid nanodomains via electrostatic interactions with anionic PIPs and leads to the formation of bigger lipid domains where protein C can interact with one another and initiate/relay a signal. There are two main domain types allowing peripheral binding of proteins, the anionic lipid and/or calcium-dependent C2 domains (such as domain A in this figure) and the anionic lipid dependent PH domains (such as domain B in this figure). **(E)** Schematic view of plant cell-to-cell junction showing the cell wall (CW), the endoplasmic reticulum (ER) network, plasma membrane (PM) and several plasmodesmata (PD). The ***right*** insert shows the PD ultrastructure. The close vicinity between the PM and the desmotubule (Dt;a lumen-free tubule of ER), connected by spoke-like tethering elements, leaves a small inter-membrane gap between the two membranes, called the cytoplasmic sleeve (CS).

There are two main elements playing a role in membrane fluidity and lipid domain formation and conservation. A very general feature is the liquid-liquid phase separation, caused by the tendency of sterols to associate with saturated lipids or proteins and form sterol-enriched ordered domains (liquid ordered Lo versus liquid disordered Ld domains) and of unsaturated lipids to tune the phase separation stability (Levental et al., 2016; Javanainen et al., 2017; Weiner and Feigenson, 2018). More precisely, in plants, a model of PM nanodomain has been proposed to involve plant-specific sphingolipids called Glycosyl Inositol Phospho Ceramides (GIPCs). GIPCs possess very long saturated acyl chains and presumably locate in the outer leaflet of the PM. Poly glycosylated GIPCs tend to increase the size of phytosterol-dependent ordered domains through cooperative interactions (Figure 1A; Grosjean et al., 2015), which likely mirrors poly phosphoinositides-enriched domains in the inner leaflet, possibly through interdigitation; i.e., interaction through very long fatty acyl chains between outer and inner leaflet lipids (Raghupathy et al., 2015; Cacas et al., 2016; Gronnier et al., 2016).

The natural segregation of lipids into domains, caused by their intrinsic properties is used, controlled and balanced by the cell through the action of proteins in order to build functional entities capable of molecular and cellular operations such as signaling (Sezgin et al., 2017). The rigidity/fluidity of the membrane partially derives from the proportion of sterols present in the bilayer, as their stiff planar structure is constraining the acyl chains of neighboring lipids (Dufourc, 2008). As a consequence, the presence of nanodomains and membrane-associated cytoskeleton is directly impacting the mobility of peripheral and anchored protein. This so-called anomalous diffusion of membrane-associated proteins and lipids could be as important as membrane compartmentalization for mesoscopic dynamics (100–1000 nm) (Wu et al., 2016). In addition, the sterol enrichment together with the orderliness and length of the lipid acyl chains are associated with the thickness of the bilayer (Javanainen et al., 2017). One example of protein sorting associated to lipid nano-domain formation is the distribution of transmembrane domains via the hydrophobic mismatch; i.e., the properties of the transmembrane domain is correlated to specific lipid domains (Figure 1B; Milovanovic et al., 2015; Lorent et al., 2017). A recent study describing the plasmodesmata proteome of *Populus trichocarpa* shows an increase in the length of the transmembrane domains of plasmodesmata-associated proteins in comparison with membrane-associated proteins (Leijon et al., 2018). This observation is in correlation with the specificity of the membrane composition described at post cytokinesis plasmodesmata (Grison et al., 2015) and pointing toward a thick “raft-like” membrane.

In animals, MCSs between the ER and the *trans-*Golgi network are critical for the regulation of the sterol and sphingolipid transfer, mediated by the Ceramide Transport Protein (CERT) and the Oxysterol Binding Protein (OSBP), which is very important for the control of *trans-*Golgi lipid composition, hence PM lipid composition (Yamaji et al., 2008; Olkkonen, 2015; Jain and Holthuis, 2017; Hanada, 2018). GIPCs being plant-specific sphingolipids, understanding their role in membranes and how they could indirectly act at MCS by modulating lipid composition would be a major step forward in cell biology. Although some studies have shown enrichment of sphingolipids and phytosterols at some plant MCSs (Fujimoto et al., 2011; Grison et al., 2015), we currently don’t know the role of inter-organellar exchange in maintaining these local lipid environments. The remaining enigma behind the role of leaflet interdigitation mediated by the GIPCs’ very long chain fatty acids and more globally the asymmetrical distribution of lipids between the inner and outer leaflets of the PM is also worth our attention (Cacas et al., 2016; Gronnier et al., 2016).

### Membrane Curvature and Lipid Packing

Another major component of the establishment of specialized membrane domains is membrane curvature and lipid packing. The latter can be described as the orderliness of the lipid arrangement: lipid packing defects arise when cavities in the membrane are formed at the interface with water, exposing aliphatic carbons (Figure 1C; Jackson et al., 2016; Gautier et al., 2018). This property of the bilayer relies upon a balance between the size of the lipid polar head and the degree of lipid unsaturation (Bigay and Antonny, 2012) but also upon the curvature of the bilayer itself (Harayama and Riezman, 2018). Other studies also suggest the formation of lipid packing defects at Lo/Ld boundaries (Tripathy et al., 2018). These membrane biophysical properties can drive membrane adsorption of various peripheral proteins which recognize lipid packing defects through, for instance, amphipathic helices in membrane curvature-sensing proteins (Cui et al., 2011; Vanni et al., 2013; Simunovic et al., 2015). In addition, the curvature itself can drive autonomous sorting of molecules depending on their properties, as it was shown for lipids (Baoukina et al., 2018) and transmembrane proteins (Aimon et al., 2014). In the context of MCSs, highly negatively curved membranes, such as PM inside plasmodesmata intercellular pores, could cluster small polar head lipids like phosphatidic acid (PA) and/or specific proteins, to potentially regulate the function of the MCS.

Other proteins or local production/degradation of specific lipids have been shown to induce membrane curvature (Tilsner et al., 2016; Choudhary et al., 2018; Ramakrishnan et al., 2018). The transmembrane region of human MCTP2 (Multiple C2 domains and Transmembrane region Protein 2), a protein that is suspected to act as a tether at EPCS in neurons (Genç et al., 2017), was notably shown to act as a reticulon domain, constraining the ER network into narrow tubules by inducing curvature (Joshi et al., 2018). An interesting question to ask is whether tether proteins can also shape membranes at MCSs and how this could be linked with inter-organellar exchange. Does the curvature induced by these tethers aim to facilitate lipid extraction for transfer? Sterol extraction could indeed be facilitated at positively curved membranes (Bigay and Antonny, 2012) and maybe more stably incorporated into membranes with no lipid packing defect such as negatively curved membranes, possibly providing a driving force for directional movement.

### Membrane Electrostatics and Ions

The third main element defining membrane and domain identity is the charge carried by the lipid polar heads, more precisely anionic lipids. In plants, phosphatidylinositol-4-phosphate (PI4P) is the major anionic lipid that drives the electrostatic identity of the PM inner leaflet (Simon et al., 2016) but a more recent research shows that the electrostatic field is actually controlled by a combination of several charged lipids, namely PI4P, PA and phosphatidylserine (PS) (Platre et al., 2018). This three-way electrostatic landscape of plant PM is critical for the creation of specific local charges and thus the recruitment and function of cationic proteins involved in cellular responses, such as the brassinosteroid transport regulator BRI1 KINASE INHIBITOR1 (BKI1) and auxin polarity modulators AGC kinases PINOID and D6-PROTEIN KINASE (D6PK) (Barbosa et al., 2016; Simon et al., 2016; Platre et al., 2018).

Negatively charged lipids are also critical elements of EPCSs, acting as co-factors for membrane tethering through direct interaction with tether proteins. Few examples are tricalbins (Tcb1-3) and Ist2 proteins in yeast (Manford et al., 2012), extended-synaptotagmins (E-Syt1-3), TMEM16, junctophilins and STIM1 in humans and finally synaptotagmin 1/A (Syt1) and MCTPs in plants (Henne et al., 2015; Tilsner et al., 2016; Brault et al., 2018). Indeed, many LTPs/tethering elements possess pleckstrin homology (PH) or C2 domains, which are known anionic lipid-interacting domains (Wong et al., 2018). In animals, MCS tether proteins presenting a series of C2 domains (like E-Syt1) were shown to have conditional environment-mediated structural modifications, which initiate or relay a signal at the MCS scale: decrease of inter-membrane gap, lipid exchange, protein complex formation/loosening (Saheki and De Camilli, 2017; Zhou et al., 2017; Bian et al., 2018). In plants, we are running late on understanding the dynamic molecular mechanisms occurring at MCSs but still, Syt1 C2 domains were shown to interact with anionic lipids (Schapire et al., 2008; Pérez-Sancho et al., 2015) and new insights on the function of MCTP family at plasmodesmata EPCS might give us some clues as their C2 domains also have the capacity to interact with PS and PI4P (Brault et al., 2018).

Local lipid modifications, pH and gradients/local concentrations of ions must also be taken into account in the regulation of the membrane electrostatic signature and thus the ability of anionic lipid-protein interactions. We know that MCSs are places of calcium exchange and anionic lipid concentration (Muallem et al., 2017). It is important to consider how the two are related and the consequences it has on MCS functions. For instance, the function of E-Syt1, which relies on the membrane docking ability of its C2 domains with anionic lipids, can be directly modulated by the presence of calcium ions (Idevall-hagren et al., 2015; Bian et al., 2018) but the latter can also shield PIP polar heads and prevent protein binding at places undergoing signaling (Seo et al., 2015; Bilkova et al., 2017; Himschoot et al., 2017; Figure 1D). Recent work has also demonstrated the effect of local concentrations of bivalent cations, mainly calcium, on the shaping of membranes containing anionic lipids: the clustering of PS and PI(4,5)P2 caused by ion interactions drives a negative curvature and tubulation of the bilayer (Doosti et al., 2017; Graber et al., 2017). A last element that is able to determine a spatiotemporal electrostatic signature is the pH, which can act on anionic lipids, mainly PA (Shin et al., 2011; Tanguy et al., 2018). It is possible that the pH at MCS could differ from the bulk cytosol and studying its variations at these areas by using pH probes could be interesting.

## Lipid Exchange at MCS

At MCS, we observe an alternative transport to vesicular trafficking: a direct shuttle/exchange of lipids between membranes. This exchange seems to be a way to guarantee robust mechanism of lipid transfer and regulation between compartments as it results in organellar lipid modifications and plays a major role in cellular events such insulin response (Lees et al., 2017) and neuronal growth (Petkovic et al., 2014). This fast and efficient crosstalk is performed by a specialized group of proteins, the lipid transfer proteins (LTPs) and relies on protein membrane binding through lipid interaction (mainly anionic lipid and/or calcium-dependent C2 domains and anionic lipid-dependent PH domains), but also on the close proximity of the two membranes (Figure 1D; Wong et al., 2018). Non-vesicular transport of lipids by LTPs is important for the regulation of membrane composition in tight places, which cannot be achieved by vesicles. It may also play an essential role in controlling the bulk lipid distribution of organelles.

For example, the OSBP and OSBP-Related Proteins (ORP, Osh) associate with vesicle-associated membrane protein-associated proteins (VAPs) at ER MCSs to specifically exchange sterols, PS and PIP molecules (Olkkonen, 2015; Moser von Filseck and Drin, 2016). Osh4 uses the PI4P imbalance created at the ER by PI4P phosphatase Sac1p to exchange PI4P extracted from the *trans-*Golgi network with sterols. This counter-flow process results in sterol enrichment at the *trans-*Golgi network and PI4P pool maintenance at the ER (Saint-jean et al., 2011). Interestingly, maintaining this PI4P pool at the ER allows the recruitment of CERT in order to transport ceramide from the ER to the *trans-*Golgi (Yamaji et al., 2008; Moser von Filseck and Drin, 2016). This trafficking of sterols and sphingolipids to the *trans-*Golgi leads to the indirect regulation of the PM lipid composition. ORP5/8 also contributes to build the PM lipid signature by counter-flowing PS to it, in exchange of PI4P and more efficiently PI(4,5)P_2_ from the ER (Chung et al., 2015; Ghai et al., 2017). Overall, it becomes clear that the transport of sterols, sphingolipids and anionic lipids is critical for the definition of membrane signature and control of lipid composition. This leads us to believe that lipid exchange at MCSs is at the basis of membrane identity by shaping their properties through the transfer of specific lipids. It also allows the creation and maintenance of lipid gradients needed for the function of molecular machineries during cellular actions. However, our knowledge on how plant lipid transfer at MCS is able to tune organellar function and respond to signaling pathways remains limited.

## MCS at Plasmodesmata, Openings on a Very Confined Space

Plasmodesmata are plant-specific channels crossing cell walls and enabling cell-to-cell communication (Brunkard and Zambryski, 2017). They are unique as they allow continuity of PM, ER and cytosol between cells (Figure 1E) and provide a direct cytosolic road for cell-to-cell molecular trafficking of metabolites, transcription factors, RNAs and calcium, and their membranes also host signaling pathways’ machineries with receptor-like proteins (Kim et al., 2005; Rutschow et al., 2011; Furuta et al., 2012; Brunkard et al., 2015; Chen et al., 2016a; Tilsner et al., 2016; Brunkard and Zambryski, 2017). New insights into the plasmodesmata ultrastructure have revealed extremely tight vicinity (down to 3 nm) between the ER and the PM inside the pores, with spoke-like tethering elements connecting the two (Figure 1E), and highlighted the plasticity of these membrane junctions during cell growth and development (Nicolas et al., 2017). To some extent, this observation leads to the re-consideration of plasmodesmata as specialized EPCS and questions the function of ER-PM contacts at plasmodesmata (Tilsner et al., 2016; Nicolas et al., 2017). While plasmodesmata are structurally related to MCSs, being sites of ER-PM contacts, we do not know if they are involved in inter-organellar communication yet. Plasmodesmata are, however, well-established sites of intercellular communication and, over the last decade, they have emerged as important signaling hubs playing a role in ever growing aspects of plant physiology. Merging these two elements results in the possibility of plasmodesmata to be a unique kind of MCS, acting as a node for both inter-organellar and cell-to-cell communication. Indeed, organelle crosstalk would clearly play a role in plasmodesmata function and local lipid transfer activity between the membranes would be conceivable since plasmodesmata are usually 500nm long channels and reaching inside the pore for vesicles is challenging, especially in mature tissues where the cell wall will be thicker.

Plasmodesmata are also singular amongst MCSs as they present a unique structural organization and membrane biophysical properties. Inside the pore, both the ER and the PM present extreme curvature, both positive and negative. So instead of two “flat” membrane segments tethered together, plasmodesmata MCS features two membrane tubes nested into each other and sitting at cell interfaces (which is neither inside the cell, neither part of the extracellular matrix). The extremely confined space between the ER and the PM (2–3 nm) is also not usual for MCSs and tight connection between the PM and cell wall components might lead us to someday talk about WALL-PM-ER MCSs.

A global view of protein population at plasmodesmata is starting to emerge (Fernandez-calvino et al., 2011; Salmon and Bayer, 2013; Kraner et al., 2017; Brault et al., 2018) and few lipidomics, showing specific lipid composition of plasmodesmata-enriched biochemical fraction, have been performed (Grison et al., 2015). However, we currently have little understanding on how the lipid and protein populations are regulating each other and how they play a role in plasmodesmata dynamics. A glimpse on the identity, structure and mode of action of plasmodesmata-associated tethering elements could open the door on understanding the molecular mechanisms taking place at plasmodesmata and potentially bridge extracellular, PM and endomembrane signaling.

## Understanding the MCS and Its Dynamics Require Interdisciplinary Approaches

Understanding the dynamics of MCSs and its actors (lipid-protein, lipid-lipid and protein-protein interactions) requires bridging across scales from atomic (or near-atomic) to cellular and tissue levels, to get a comprehensive picture of MCSs. While cellular and tissue-level events can be tackled by classical cell biology (such as confocal microscopy) and genetic tools, their limits in terms of resolution encourage the use of *in silico,* biophysical-based tools and electron microscopy for understanding MCSs at atomic/macromolecular-levels. Many options are possible but a number of approaches are especially interesting in the context of protein/lipid interaction, hence MCSs (see Table 1). For example, molecular modeling and dynamic simulations are relatively easy-accessible ways to study, simultaneously or not, the structure and function of proteins and lipid bilayers at a molecular/atomic level and often bring evidences on questions that could not be answered by other means (Javanainen et al., 2017). Currently, the increasing computational power and the development of efficient coarse grained force fields for an increasing number of molecules^1^ (Marrink et al., 2007) allow the simulation of bigger and more complex systems during longer time scales (up to the micro-scale) (Duncan et al., 2017; Hsu et al., 2017), which fit MCS scales.

**Table 1:** Non-extensive list of tools usable for atomic/macromolecular-level study of MCSs.

Technique	Usage	Reference
*In silico*		
Hypermatrix	Energy-based calculation of lipid-ligand interactions and 3D arrangements	Deleu et al., 2014; Cacas et al., 2016
IMPALA	Energy-based prediction of the insertion of molecules in lipid bilayers	Basyn et al., 2001; Lins et al., 2001; Cacas et al., 2016
Molecular dynamics	Atomic and coarse grained simulations to study the behavior over time of lipids bilayers and proteins	Deleu et al., 2014; Yamamoto et al., 2016; Duncan et al., 2017; Gronnier et al., 2017
*In vitro*		
PIP Strips	Determination of protein ability to interact with specific anionic lipids	Pérez-Sancho et al., 2016
Liposome flottation/sedimentation assays	Determination of protein ability to interact with a lipid bilayer	Schapire et al., 2008; Pérez-Sancho et al., 2016; Meca et al., 2018
Tubule formation by optical tweezers on liposome	Study of membrane curvature-induced sorting of proteins	Aimon et al., 2014; Prévost et al., 2015; Chen et al., 2016b
*In vitro* tethering to reconstitute simplified MCS with isolated protein and controlled lipid and ion environment.	Characterization of the ability of a protein to tether two liposomes using dynamic light scattering and the inter-liposome distance by FRET. Visualize the tethering ultrastructure using cryo-electron microscopy	Mesmin et al., 2013; Lin et al., 2014; Diao et al., 2015
Isothermal Titration Calorimetry (ITC)	Determination of the affinity constant and thermodynamics parameters for the interaction between proteins and liposomes.	Ghai et al., 2012
Langmuir Trough	Determination of the kinetics of adsorption and affinity parameters of proteins for lipid monolayers	Eeman et al., 2006; Calvez et al., 2011; Gronnier et al., 2017
Solid state NMR	Study lipid-protein interactions and the deformation of the lipid membrane caused by the interaction at atomic level	Huster, 2014; Gronnier et al., 2017
*In situ*		
(Cryo) electron tomography	Visualize MCS architecture at macromolecular scale	Collado and Fernández-Busnadiego, 2017; Nicolas et al., 2017


The study of a system closely related to MCSs, the SNARE (Soluble NFS attachment protein receptor)-mediated membrane fusion, involved for example in the highly regulated release of neurotransmitters at the synapse in animals (Chen and Scheller, 2001), proves the need for multidisciplinary tools to understand the molecular operations and underlying subtleties. Animal synaptotagmin 1 (Syt1), a tether protein that possesses a transmembrane domain and two C2 domains, is a major actor of SNARE as it is implicated in each step of the neurotransmitter release process. For example, the role of PIP, PS and calcium in PM docking of Syt1 C2 domains and bridging of the membranes was revealed by using isothermal titration calorimetry (ITC), fluorescence energy transfer (FRET) and vesicle sedimentation assays, NMR and computational modeling (Lin et al., 2014; Pérez-Lara et al., 2016). Understanding the causes and function of the ring-like oligomerization of Syt1and the role of tandem C2 domain interaction was performed using electron microscopy, circular dichroism, ITC, atomic force microscopy, floatation, and sedimentation assays (Evans et al., 2016; Zanetti et al., 2016). Comprehending the nature of the Syt1-SNARE complex interaction was possible mainly through NMR and molecular modeling and dynamics (Brewer et al., 2015). All these techniques brought an integrated vision of the dynamic molecular mechanisms occurring at this crucial interface. We believe that employing similar resources for MCS-associated processes would undoubtedly bring us new and original insights in these peculiar systems of cell biology.

## Conclusion

There is still a lot to be done in the understanding of plant EPCS function and the molecular mechanisms involved in their dynamics and regulation. Important questions concern the function and role of membrane compartmentalization (lipid nanodomains, inner/outer leaflet composition, interdigitation), the molecular mechanisms associated with the tethering machinery at MCSs (tethers’ identity, effect of tethering in lipid transfer and signaling pathways) and the roles of the lipid environment in the definition of MCSs (regulation, dynamics). However, increasing technical resources have helped to grasp pieces of the puzzle that we are only now starting to assemble. The complexity arising from the incredible diversity in lipids and proteins and, over all, the complex relationships that interconnect them are not making the task easy to accomplish. The biophysical properties of the membrane derived from the intrinsic nature of a plethora of lipids species and their mutual interactions, is impacting on the recruitment and function of proteins, which in turn are fine tuning their lipid environment. The effects of this cycle are expected to get even more intertwined inside very confined environments, such as MCSs, and the entanglement is such that every molecule and every interaction is part of the dance, driving short or long-term consequences on MCS function.

## Author Contributions

JP did the writing, figure, and table. FI and LL did the corrections and advised on the manuscript content. EB supervised the writing and did the corrections and comments.

## Conflict of Interest Statement

The authors declare that the research was conducted in the absence of any commercial or financial relationships that could be construed as a potential conflict of interest.

## Footnotes

1 http://cgmartini.nl/
